# ZFX acts as a transcriptional activator in multiple types of human tumors by binding downstream from transcription start sites at the majority of CpG island promoters

**DOI:** 10.1101/gr.228809.117

**Published:** 2018-03

**Authors:** Suhn Kyong Rhie, Lijun Yao, Zhifei Luo, Heather Witt, Shannon Schreiner, Yu Guo, Andrew A. Perez, Peggy J. Farnham

**Affiliations:** Department of Biochemistry and Molecular Medicine and the Norris Comprehensive Cancer Center, Keck School of Medicine, University of Southern California, Los Angeles, California 90089, USA

## Abstract

High expression of the transcription factor ZFX is correlated with proliferation, tumorigenesis, and patient survival in multiple types of human cancers. However, the mechanism by which ZFX influences transcriptional regulation has not been determined. We performed ChIP-seq in four cancer cell lines (representing kidney, colon, prostate, and breast cancers) to identify ZFX binding sites throughout the human genome. We identified roughly 9000 ZFX binding sites and found that most of the sites are in CpG island promoters. Moreover, genes with promoters bound by ZFX are expressed at higher levels than genes with promoters not bound by ZFX. To determine if ZFX contributes to regulation of the promoters to which it is bound, we performed RNA-seq analysis after knockdown of ZFX by siRNA in prostate and breast cancer cells. Many genes with promoters bound by ZFX were down-regulated upon ZFX knockdown, supporting the hypothesis that ZFX acts as a transcriptional activator. Surprisingly, ZFX binds at +240 bp downstream from the TSS of the responsive promoters. Using Nucleosome Occupancy and Methylome Sequencing (NOMe-seq), we show that ZFX binds between the open chromatin region at the TSS and the first downstream nucleosome, suggesting that ZFX may play a critical role in promoter architecture. We have also shown that a closely related zinc finger protein ZNF711 has a similar binding pattern at CpG island promoters, but ZNF711 may play a subordinate role to ZFX. This functional characterization of ZFX provides important new insights into transcription, chromatin structure, and the regulation of the cancer transcriptome.

Altered transcriptomes are a general characteristic of human cancers. In many cases, the transcriptional dysregulation is driven by altered expression levels or activity of transcription factors (TFs) (Yao et al. 2015; Rhie et al. 2016). There are about 2000 DNA-binding TFs in the human genome, but little is known about most of these regulators (Vaquerizas et al. 2009; Wingender et al. 2015). We previously identified distinct sets of TFs having increased expression associated with different cancers (Yao et al. 2015; Rhie et al. 2016). In contrast, ZFX, a zinc finger protein (ZNF) that contains a DNA binding domain, has been implicated in the initiation or progression of many different types of human cancers, including prostate cancer, breast cancer, colorectal cancer, renal cell carcinoma, glioma, gastric cancer, gallbladder adenocarcinoma, non-small cell lung carcinoma, and laryngeal squamous cell carcinoma (Zhou et al. 2011; Fang et al. 2012; Jiang et al. 2012b,c; Nikpour et al. 2012; Li et al. 2013; Fang et al. 2014a,b; Yang et al. 2014; Weng et al. 2015). In these previous studies, it was shown that high expression of ZFX is linked to tumorigenesis, and knocking down ZFX can suppress cellular proliferation and increase the proportion of apoptotic cells (Fang et al. 2014a; Jiang and Liu 2015; Yang et al. 2015; Yan et al. 2016). In addition, high ZFX expression correlates with poor survival of cancer patients (Jiang and Liu 2015; Li et al. 2015; Yang et al. 2015; Yan et al. 2016). For example, ZFX expression is significantly related to histological grade (*P*-value <0.001) in gallbladder adenocarcinoma, and patients that survived <1 yr were found to have significantly higher ZFX expression than patients that survived >1 yr (Weng et al. 2015). Taken together, these studies suggest that ZFX may function as an oncogene. However, the mechanism by which ZFX may influence transcriptional regulation in such a diverse set of human tumors has not been determined.

ZFX is encoded on the X Chromosome and highly conserved in vertebrates. Among the roughly 2000 site-specific DNA-binding TFs, the C2H2 ZNFs are the largest class encoded in the human genome. Although the biological functions of the majority of ZNFs are unknown, the molecular functions of ZNFs include not only sequence-specific binding to DNA but also protein–protein interactions and RNA binding (Stubbs et al. 2011; Najafabadi et al. 2015). DNA-binding ZNFs generally have multiple, adjacent, properly spaced zinc fingers in their DNA binding domain; ZNFs with fewer than three properly spaced fingers are more likely to be involved in protein–protein or protein–RNA interactions (Brown 2005; Brayer and Segal 2008). ZFX has 13 C2H2-type zinc fingers in its putative DNA binding domain, the last nine of which are properly spaced, supporting the hypothesis that ZFX is a DNA binding factor. ZFX contains a large acidic activation domain in addition to the C2H2-type zinc finger-containing DNA binding domain, suggesting that, in contrast to the hundreds of ZNFs that contain a KRAB repression domain, ZFX may be a transcriptional activator.

Although the structure of ZFX suggests that it is a DNA binding transcriptional activator that is expressed at high levels in many different types of cancers, ZFX binding sites have not yet been mapped in cancer cells. To understand the mechanism by which ZFX may regulate the cancer transcriptome, we performed ChIP-seq, NOMe-seq, and RNA-seq assays with knockdown experiments in HEK293T kidney, HCT116 colon, C4-2B prostate, and MCF-7 breast cancer cells, identifying ZFX-binding sites and ZFX-regulated genes throughout the human genome.

## Results

### ZFX binds to CpG island promoter regions

**Figure 1.**
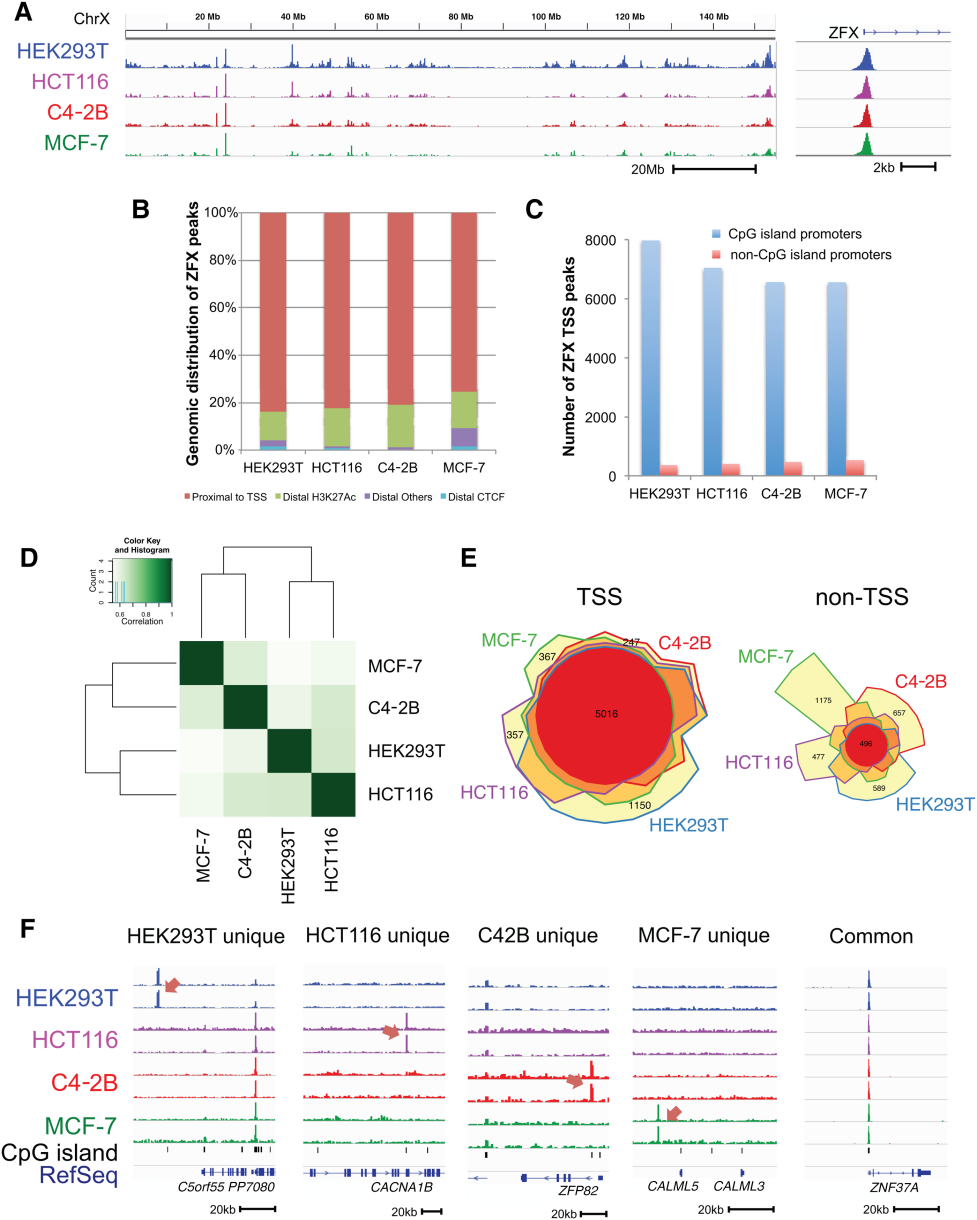
Genome-wide ZFX binding profiles in multiple types of human tumors. (*A*) ZFX ChIP-seq data for a region of ∼150 Mb of Chromosome X for HEK293T kidney, HCT116 colon, C4-2B prostate, and MCF-7 breast cancer cells (*left*) and for a region of ∼6 kb near the ZFX promoter (*right*). (*B*) The percentage of ZFX binding sites in promoters (±2 kb from the TSS), distal enhancers (H3K27ac), distal insulators (CTCF not in enhancers), and at other locations is shown for ZFX ChIP-seq data for four cell types. (*C*) The number of ZFX binding sites located in CpG island promoters versus non-CpG island promoters is shown for the four cell types. (*D*) Heatmap of the ChIP-seq signal correlation for ZFX binding sites in the four tumor types. (*E*) Venn diagrams of ZFX binding sites near promoters (*left*) and distal regions (*right*) for HEK293T kidney, HCT116 colon, C4-2B prostate, and MCF-7 breast cancer cells. (*F*) Examples of cell-type–specific and common ZFX binding sites.

To profile the genome-wide binding sites of ZFX, we performed two biological ZFX ChIP-seq replicates using chromatin from HEK293T kidney, HCT116 colon, C4-2B prostate, and MCF-7 breast cancer cells (Fig. 1A; see Supplemental Fig. S1 for ZFX antibody validation and Supplemental Table S1 for access information for all genomic data sets). We chose to use these cancer models because there is a strong link in these four cancer types between ZFX expression and cell proliferation, tumor development, or patient survival. For example, prostate cancer tissues exhibit significantly higher ZFX expression than benign prostatic hyperplasia and adjacent tissues and siRNA-mediated knockdown of ZFX suppresses the proliferation of prostate cancer cells and reduces the number of colonies in colony forming assays (Jiang et al. 2012a). Similarly, expression of ZFX is high in advanced invasive breast cancers and knockdown of ZFX reduces the proliferation rate of breast cancer cells (Yang et al. 2014). High expression of ZFX promotes tumor growth of colon cancer cells and colorectal cancer patients with high ZFX expression have poorer overall and disease-free survival (Jiang and Liu 2015). Moreover, knockdown of ZFX suppresses proliferation and invasion of colon cancer cell lines (Jiang and Liu 2015). Finally, ZFX is significantly up-regulated in renal cell carcinomas (RCC) and has been suggested to be a strong predictor for prognosis of RCC patients (Li et al. 2015). Also, knockdown of ZFX expression in renal carcinoma cells results in significantly inhibited proliferation and cell cycle progression (Fang et al. 2014a).

We identified roughly 9000 reproducible ZFX binding sites in each cancer cell line (HEK293T: 9955; HCT116: 9039; C4-2B: 8708; MCF-7: 9382). Annotation of the ZFX binding sites with respect to different genomic regions showed that ∼80% of the sites are located in a promoter region (±2 kb of a TSS). In each of the cell types examined, only about 1000 sites are located in distal elements (i.e., distal sites having H3K27ac or CTCF peaks or other distal sites that are not marked with H3K27ac or CTCF) (Fig. 1B). To further classify the promoter binding sites, we determined whether ZFX preferentially binds to housekeeping, CpG island promoters, or to more cell-type–specific, non-CpG island promoters. We found that the majority of the ZFX peaks are in CpG island promoters (Fig. 1C). In fact, we identified more than 13,000 CpG island promoters that are bound by ZFX in the union of the four cell lines, with ∼60% of all active CpG island promoters in a given cell type being bound by ZFX, including a strong peak at the promoter of the ZFX gene (Fig. 1A, right; Supplemental Tables S2, S3A–D).

### The ZFX binding pattern at promoter regions is very similar in different cancer types

Many oncogenic TFs bind to distinct cell-type–specific distal regulatory elements in different types of tumors (Rhie et al. 2016). However, the majority of ZFX peaks are in promoter regions, suggesting that ZFX may bind to regulatory elements that are common to all cell types, rather than to cell-type–specific regulatory elements. To further analyze the ZFX binding patterns, we compared the ZFX binding sites in the four cancer cell lines; the binding patterns are similar to each other in general (correlation coefficient >0.5) (Fig. 1D). We then separated the peaks into TSS proximal and non-TSS (>2 kb from a TSS). We found that the ZFX binding sites in promoter regions are largely shared among the four cell lines, with ZFX commonly binding to more than 5000 promoter peaks in all four cell lines (Fig. 1E; Supplemental Table S3E). In contrast, the distal sites bound by ZFX are not always the same in the different cell types. We note that both the common and the cell-type–specific ZFX binding sites are robust and reproducible (Fig. 1F).

### ZFX motifs are enriched at 240 bp upstream of and downstream from the TSS in CpG island promoters, but ZFX prefers to bind downstream from the TSS

**Figure 2.**
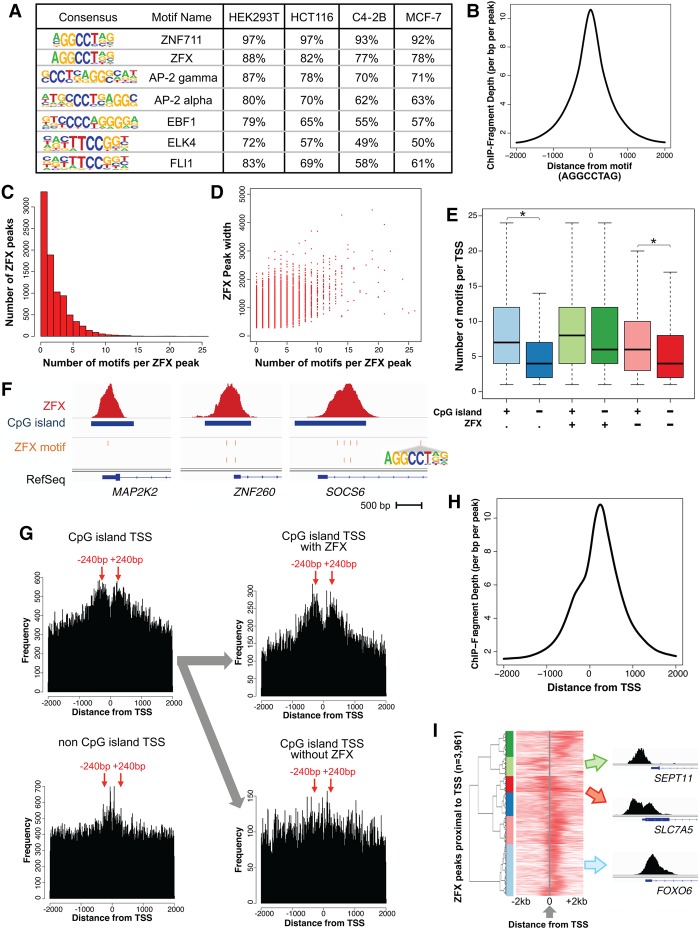
Characterization of ZFX motifs and binding sites. (*A*) Motifs enriched at summits of the ZFX binding sites and the percentage of ZFX peaks having each motif for HEK293T kidney, HCT116 colon, C4-2B prostate, and MCF-7 breast cancer cells; the same motifs were identified in the set of ZFX peaks located near a TSS and the set of distal ZFX peaks. (*B*) Average ZFX ChIP-seq signal in C4-2B relative to ±2 kb from the motif AGGCCTAG. (*C*) Histogram of the number of motifs per ZFX peak. (*D*) Scatterplot of the relationship between the number of motifs per ZFX peak and ZFX peak width. (*E*) Number of AGGCCTAG motifs per TSS region (±2 kb from the TSS) for different groups of promoters: (light blue) CpG island promoters; (blue) non-CpG island promoters; (light green) CpG island promoters bound by ZFX; (green) non-CpG island promoters bound by ZFX; (light pink) CpG island promoters not bound by ZFX; (red) non-CpG island promoters not bound by ZFX. Comparisons of data sets that show a significant difference (adjusted *P*-value <0.05) are marked with an asterisk. (*F*) Example of ZFX binding site and motif position at CpG island promoters having one (*MAP2K2*) or more (*ZNF260*, *SOCS6*) motifs. (*G*) Frequency of AGGCCTAG motifs located ±2 kb from the TSS of CpG island promoters, of non-CpG island promoters, of CpG island promoters bound by ZFX, and of CpG island promoters not bound by ZFX; a comparison to results obtained using a scrambled motif can be found in Supplemental Figure S2. (*H*) Average ZFX ChIP-seq signal ±2 kb from the TSS of promoters bound by ZFX in MCF-7. (*I*) Heatmap representing unsupervised clustering of ZFX ChIP-seq signals in MCF-7 cells at promoters bound by ZFX that only have one TSS in a ±2 kb region (*n* = 3961). Also shown are examples of ZFX binding sites from the light green cluster (promoters having a ZFX peak only upstream of the TSS), the red cluster (promoters having a ZFX peak both up and downstream from the TSS), and the light blue cluster (promoters having a ZFX peak only downstream from the TSS). Genes from each cluster are listed in Supplemental Table S3F.

To determine the preferred binding motif for ZFX, we performed motif analyses using 20-bp windows from ZFX peak summits. ZFX has nine properly spaced zinc fingers; because a zinc finger can bind to 3 nt of DNA (Desjarlais and Berg 1992), one would expect a 27-nt motif if all of these fingers are involved in DNA binding. However, the motif we identified in the majority of the ZFX peaks was only 8 nt (AGGCCTAG) with a strong 6-nt consensus (AGGCCT) (Fig. 2A). This motif was originally identified from ZNF711 ChIP-seq data from the brain tumor cell line SH-SY5Y (Kleine-Kohlbrecher et al. 2010) and from ZFX ChIP-seq data from mouse embryonic stem cells (Chen et al. 2008). We also identified several other CT-containing motifs, such as the motif for AP-2. It is possible that multiple sets of zinc fingers in ZFX may recognize a repeating unit of a short motif. Alternatively, it is possible that ZFX utilizes only a subset of its fingers to bind DNA; we have previously described this situation for two artificial six-finger ZNFs (Grimmer et al. 2014). To further analyze the ZFX binding preferences, we focused on the AGGCCTAG motif, which is found in >90% of the ZFX peaks in each cell line. We showed that the motif is centered in the ZFX peaks, suggesting that this motif directly recruits ZFX (Fig. 2B). However, we also found that some ZFX peaks have many copies of the motif (Fig. 2C), with larger peaks having, in general, more copies (Fig. 2D). Next, we asked whether this motif is found in all promoter regions or only in promoters bound by ZFX. We found that 48,373 of 57,820 promoters of all known genes in the human genome have at least one copy of this motif ±2 kb from the TSS. In fact, there are multiple copies of this motif in most promoter regions, with more motifs in CpG island promoters (Fig. 2E). We note that promoters bound by ZFX have, on average, slightly more motifs than promoters not bound by ZFX. However, the number of motifs does not necessarily correspond to the number of ZFX peaks in a promoter. Some promoters, like *MAP2K2*, have one motif and one binding site. Other promoters, such as *ZNF260* and *SOCS6*, have multiple copies of the motif, but not all of the motifs are bound by ZFX (Fig. 2F).

We further investigated the distribution of the AGGCCTAG motif relative to the TSS. We found that this motif is symmetrically enriched ±240 bp from the TSS, with CpG island promoters showing greater enrichment than non-CpG island promoters and CpG island promoters bound by ZFX showing greater enrichment than those not bound by ZFX (Fig. 2G). The symmetrical distribution of the motif ±240 bp relative to the TSS is unusual and specific for the ZFX motif and not scrambled variants (Supplemental Fig. S2). Therefore, we asked whether ZFX binding has a similar distribution or, as for most TFs, if ZFX binds mainly upstream of the TSS. We found that ZFX has a stronger preference for binding at +240 bp downstream from the TSS (Fig. 2H). The reason that there are some peaks at −240 bp could be due to the inclusion of bidirectional promoters. To test this hypothesis, we selected the ZFX binding sites that have only one known TSS within a ±2 kb window (*n* = 3961) and plotted heatmaps centered to each TSS (Fig. 2I; Supplemental Table S3F). Most of the time, ZFX is bound at +240 bp of the TSS (e.g., *FBXO6*). However, there are a small number of promoters (<10%) that have a ZFX peak at −240 bp (e.g., *SEPT11*), and some promoters that have two ZFX peaks symmetrically located on either side of the TSS (e.g., *SLC7A5*). We conclude that the presence of motif, which is symmetrically enriched ±240 bp from the TSS may be necessary, but is not sufficient, to recruit ZFX because ZFX has a higher binding frequency at +240 bp than at −240 bp.

### ZFX has properties of a transcriptional activator

**Figure 3.**
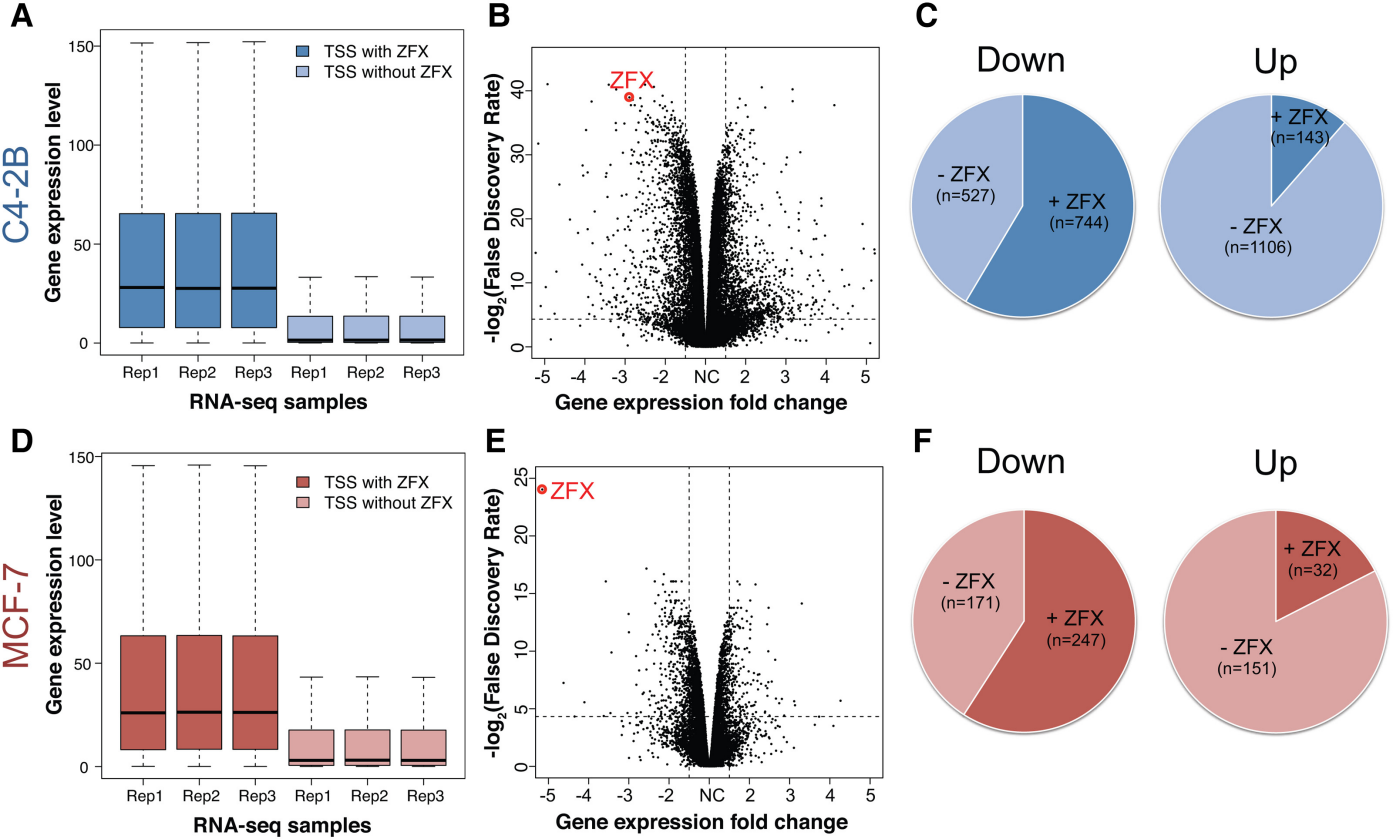
The role of ZFX in transcription regulation. Expression levels of genes with active promoters bound by ZFX and genes with active promoters not bound by ZFX are shown for C4-2B (*A*) and MCF-7 (*D*); active promoters are defined by detectable expression of a transcript from that promoter in that particular cell line. Volcano plots demonstrate differential gene expression after knockdown of ZFX in C4-2B (*B*) and MCF-7 (*E*). Comparisons of the percentage of down-regulated versus up-regulated genes that have ZFX bound at their promoters are shown for C4-2B (*C*) and MCF-7 (*F*) cells.

To determine if ZFX functions as a transcriptional activator or repressor, we separately analyzed expression levels of genes regulated by promoters that are bound by ZFX versus those promoters not bound by ZFX. We found that the median expression level of genes with promoters bound by ZFX is much higher than the median expression level of genes with promoters not bound by ZFX (Fig. 3A), suggesting that ZFX may be a transcriptional activator. To gain insight into possible mechanisms by which ZFX might activate transcription, we used transient transfection with siRNA to knock down the levels of ZFX in C4-2B prostate cancer cells and identified 1271 genes whose expression decreased and 1249 genes whose expression increased upon reduction of ZFX levels in C4-2B cells (FDR <0.05, fold change >1.5) (Fig. 3B; Supplemental Table S4A). Genes identified as responsive to changes in the level of a TF include both direct target genes and genes that are in downstream signaling pathways regulated by the direct target genes (i.e., indirect targets). One approach to identify direct ZFX target genes is to determine which of the deregulated genes have ZFX binding sites in their promoter regions. We found that the promoters of 744 of the 1271 down-regulated genes (58.5%), but only 143 promoters of the 1249 up-regulated genes (11.4%) are bound by ZFX in C4-2B cells (Fig. 3C).

We repeated the siRNA experiments in MCF-7 breast cancer cells. Again, we found that genes having a bound ZFX in their promoter had a median higher expression level than genes without a bound ZFX (Fig. 3D). However, when we knocked down ZFX, we identified only 418 genes whose expression decreased and 183 genes whose expression increased (Fig. 3E; Supplemental Table S4B). Although the number of deregulated genes in the MCF-7 knockdown experiments is less than in the C4-2B knockdown experiments, the down-regulated genes again have a higher percentage of promoter-bound ZFX (59.1%) than do the up-regulated genes (17.5%) (Fig. 3F).

**Figure 4.**
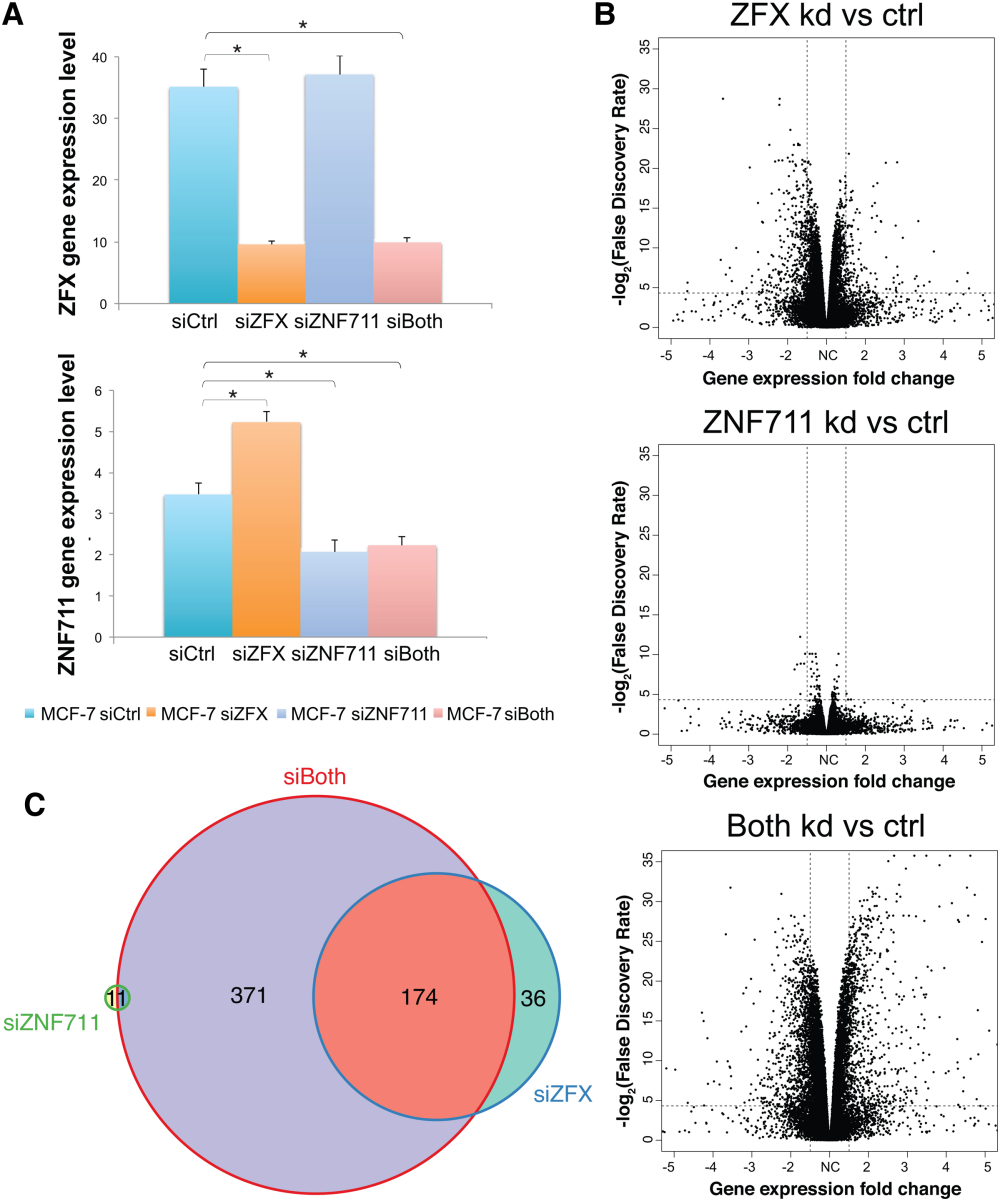
Combinatorial knockdown of ZFX and ZNF711 in MCF-7 cells. (*A*) ZFX and ZNF711 expression levels upon knockdown of ZFX, ZNF711, or both TFs in MCF-7. Comparisons of data sets that show a significant difference are marked with an asterisk (FDR <0.05). (*B*) Volcano plots demonstrating differential gene expression after knockdown (kd) of ZFX, ZNF711, or both TFs. (*C*) Comparison of down-regulated genes having ZFX bound at their promoters after knockdown of ZFX, ZNF711, or both TFs.

One explanation for the smaller effect on the transcriptome of MCF-7 cells could be inefficient knockdown of ZFX. However, the reduction in ZFX was similar in the siRNA-treated C4-2B and MCF-7 cells (Supplemental Fig. S3A). An alternative explanation could be that another TF is functionally redundant with ZFX in MCF-7. C2H2 ZNFs comprise the largest class of site-specific DNA-binding proteins encoded in the human genome and have arisen through gene duplication followed by mutation. Specifically, ZFX is very similar to ZFY and ZNF711 (Supplemental Fig. S3B); ZFX and ZNF711 are both encoded on the X Chromosome, whereas ZFY is located on the Y Chromosome. Overall protein homology is 92% between ZFX and ZFY, with the zinc finger domains having 97% homology, suggesting that these two proteins may have fully redundant activities (Schreiber et al. 2014). However, MCF-7 are female breast cancer cells and thus do not express ZFY (although C4-2B are male, they also do not express ZFY). There is 55% identity between the entire ZFX and ZNF711 proteins, with the zinc finger domains having 87% identity, also suggesting that these two TFs may have similar functions. ZNF711 is not expressed in C4-2B, but it is expressed in MCF-7 with the expression slightly increasing upon knockdown of ZFX (Supplemental Fig. S3C). To investigate the possible functional redundancy of these two TFs, in an independent set of siRNA experiments than shown in Figure 3, we knocked down ZFX, ZNF711, or both TFs simultaneously in MCF-7 (Supplemental Table S4C). We again observed an increase in ZNF711 expression when ZFX was knocked down (Fig. 4A). Several thousand genes changed upon knockdown of ZFX, but very few genes changed upon knockdown of ZNF711. However, we detected more differentially expressed genes (*n* = 1847, FDR <0.05, fold change >1.5) upon knockdown of both ZFX and ZNF711 in MCF-7 cells than in the combined single knockdown experiments (Fig. 4B). In the double knockdown, we identified 371 additional down-regulated genes that have ZFX bound to their promoters in MCF-7 cells (Fig. 4C). These findings suggest that ZNF711 may substitute for ZFX when ZFX expression is reduced by knockdown in MCF-7 cells; similar results were found when ZNF711 and ZFX were knocked down in HEK293T cells (Supplemental Fig. S4).

**Figure 5.**
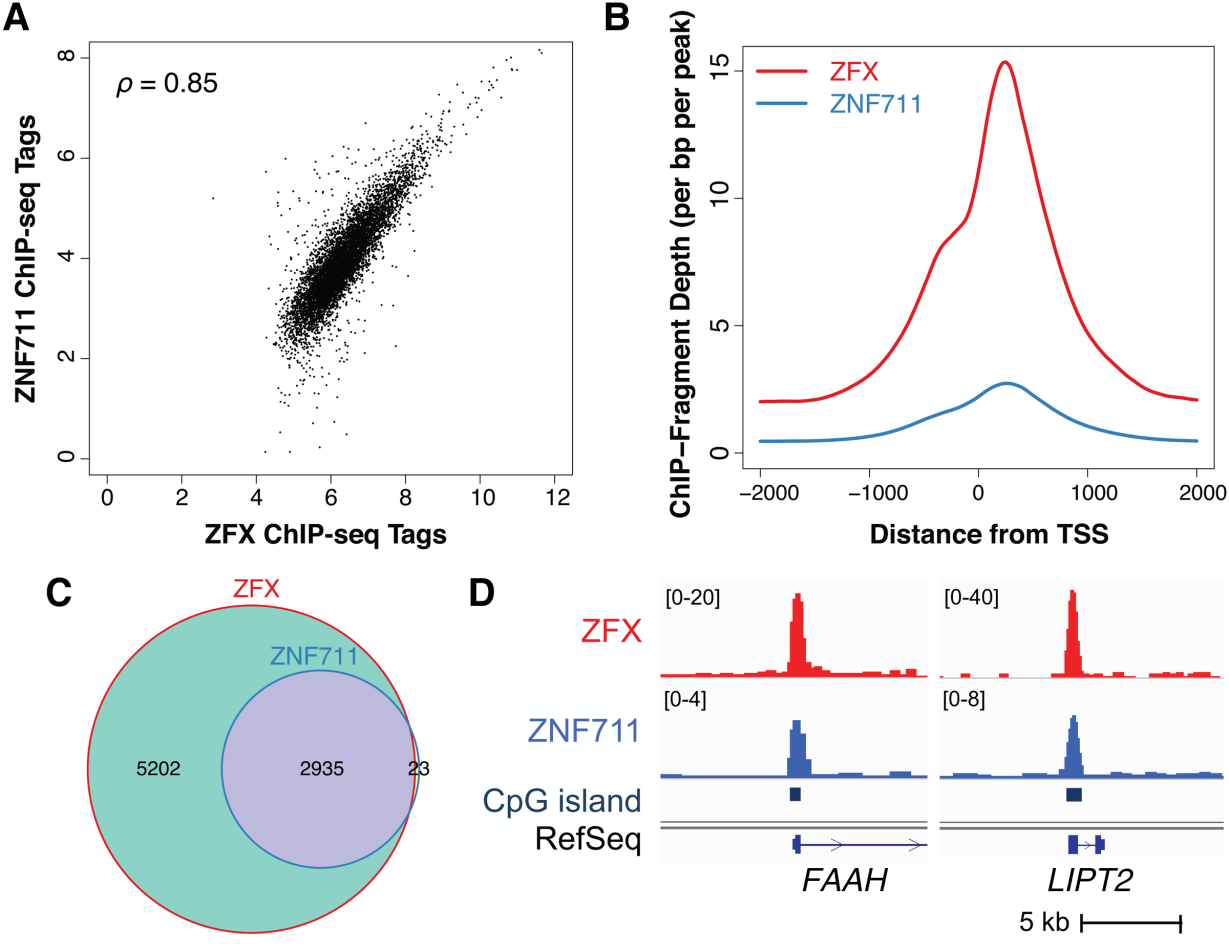
Comparison of ZFX and ZNF711 binding at promoters in MCF-7 cells. (*A*) Scatterplot of the normalized ZFX versus ZNF711 ChIP-seq tags for the union set of ZFX and ZNF711 binding sites found in promoters (ρ = 0.85, Spearman's rank correlation coefficient). (*B*) Average ZFX (red) and ZNF711 (blue) ChIP-seq signal ±2 kb from the TSS of promoters bound by ZNF711 in MCF-7. (*C*) Comparison of expressed genes having ZFX or ZNF711 bound at their promoters in MCF-7. (*D*) Examples of ZFX and ZNF711 binding at CpG island promoters for two genes down-regulated upon knockdown of both ZFX and ZNF711 in MCF-7.

To gain further support for the hypothesis that ZFX and ZNF711 are functionally redundant, we performed ChIP-seq for ZNF711 in MCF-7 cells. We identified 2708 ZNF711 binding sites genome-wide (Supplemental Table S5), 98.6% of which overlapped with ZFX binding sites in MCF-7 cells. As expected, ZNF711 binding sites were also enriched at CpG island promoter regions, both in MCF-7 cells and in SH-SY5Y cells (Supplemental Fig. S5A,B). Unlike the ZFX binding pattern in the four cancer cell types, ZNF711 appears to have more cell-type–specific binding sites (Supplemental Fig. S5). A comparison of ChIP-seq tags shows enrichment of ZNF711 at the promoter regions bound by ZFX, but the ZNF711 ChIP-seq signals are weaker than the ZFX ChIP-seq signals in MCF-7 cells (Fig. 5A), perhaps due to differences in expression levels of the two TFs (see Fig. 4A). Importantly, the ZNF711 binding sites are also enriched at +240 bp downstream from the TSS (Fig. 5B). A comparison of the set of ZNF711-bound active promoters to the set of ZFX-bound active promoters revealed that 99% of promoters bound by ZNF711 are also bound by ZFX (Fig. 5C). This binding site redundancy supports the hypothesis that ZNF711 can substitute for ZFX when ZFX is knocked down. For example, *FAAH* and *LIPT2* show statistically significantly reduced expression in the double knockdown cells (FDR <0.05) but not in the single knockdown cells, and the promoters of the *FAAH* and *LIPT2* genes are bound by both ZFX and ZNF711 in MCF-7 cells (Fig. 5D).

### ZFX binds adjacent to the first phased nucleosome downstream from the TSS

**Figure 6.**
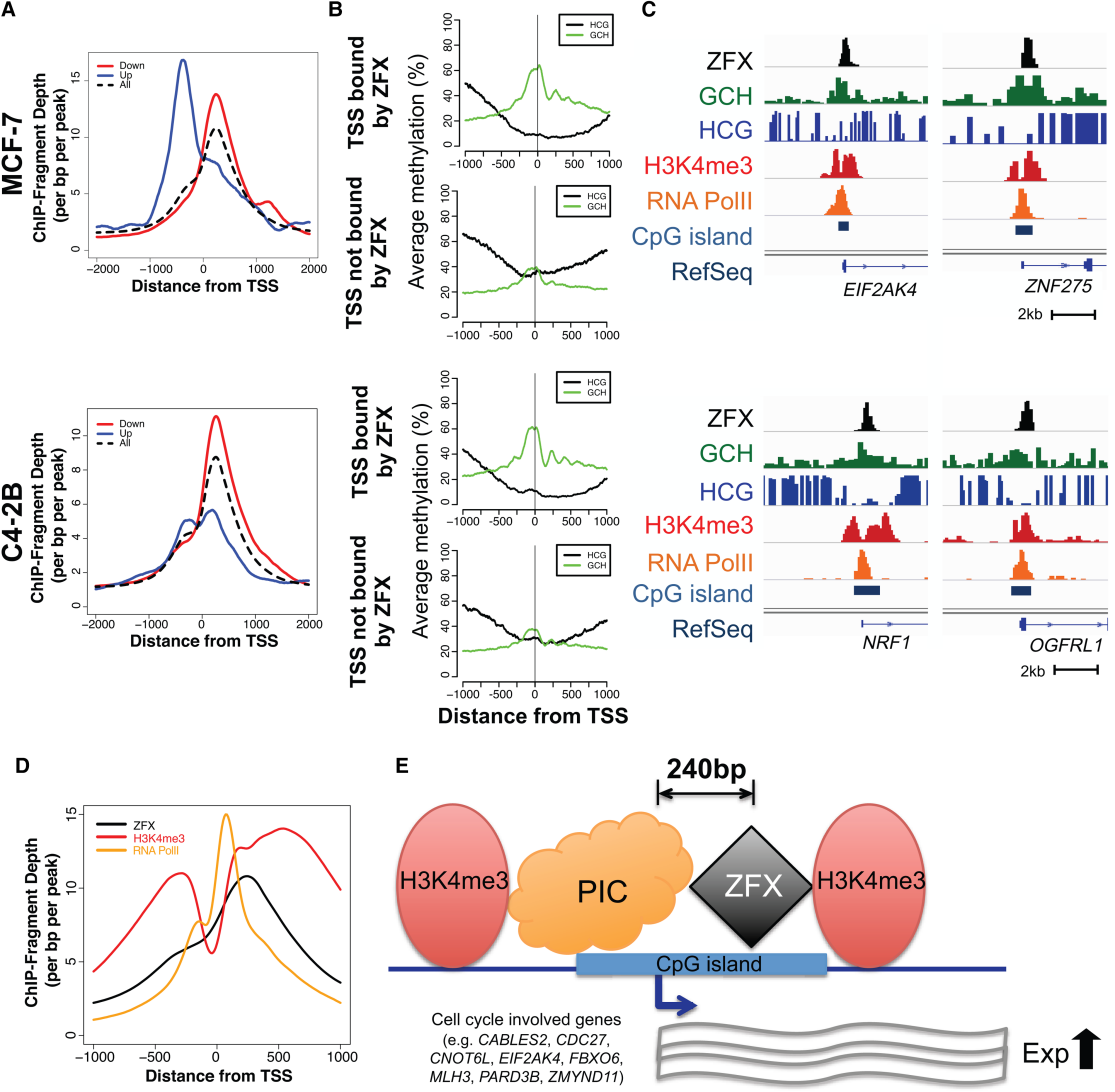
The relationship between ZFX binding and chromatin structure at promoters. (*A*) Average ZFX ChIP-seq signals ±2 kb from the TSS of down-regulated (red), up-regulated (blue), and all (black) genes bound by ZFX in MCF-7 (*top*) and C4-2B (*bottom*). (*B*) Endogenous DNA methylation (HCG) (black) and the accessibility (GCH) (green) levels from NOMe-seq data ±1 kb from the TSS of active promoters bound by ZFX and from the TSS of active promoters not bound by ZFX in MCF-7 (*top*) and C4-2B (*bottom*). (*C*) Examples of ZFX binding sites with ZFX ChIP-seq, NOMe-seq, H3K4me3 ChIP-seq, and RNA Polymerase II ChIP-seq signals in MCF-7 (*top*) and C4-2B (*bottom*). (*D*) Average ZFX (black), H3K4me3 (red), and RNA Polymerase II (orange) ChIP-seq signals ±1 kb from the TSS of genes bound by ZFX in MCF-7. (*E*) A model demonstrating the relationship of ZFX to other components of CpG island promoter structure. ZFX binds at +240 bp in the nucleosome-depleted region of CpG island promoters, between the general transcription preinitiation complex (PIC) and the first nucleosome in the transcribed region. When ZFX is bound to this downstream site, it increases the expression levels of genes involved in cell proliferation; the wavy lines represent RNA levels.

As shown above, ZFX motifs are enriched at 240 bp both upstream of and downstream from the TSS, but the majority of ZFX peaks are located at +240 bp. However, it was possible that the promoters activated by ZFX have a distinct binding pattern compared to all ZFX peaks. Therefore, we compared ZFX binding patterns at promoters of all expressed genes versus genes down-regulated or up-regulated upon ZFX knockdown (Fig. 6A). Interestingly, the down-regulated genes (genes that may be directly activated by ZFX) show a nicely positioned ZFX bound at +240 bp. In contrast, the up-regulated genes (genes that may be repressed or indirectly regulated by ZFX) are not as highly enriched for ZFX at the +240 bp position; rather there were more peaks at the −240 bp position for up-regulated genes (especially in MCF-7 cells). Taken together, these results suggest that ZFX may be a positive activator only when bound at +240 bp of the TSS.

The preferred location of ZFX at +240 bp is a unique position for a DNA binding TF. To further characterize the relationship between the bound ZFX and open chromatin surrounding the TSS, we used Nucleosome Occupancy and Methylome Sequencing (NOMe-seq). This genome-wide method identifies nucleosome-depleted regions (NDRs) and provides single molecule resolution for both accessibility and DNA methylation, which can very precisely identify specific TF binding sites (Kelly et al. 2012). When we used NOMe-seq to profile accessibility and DNA methylation in MCF-7 and C4-2B cells, we found that promoters bound by ZFX have a more accessible region with lower levels of DNA methylation near the TSS and more highly phased nucleosomes downstream from the TSS compared to promoters that are active in those cells but not bound by ZFX (Fig. 6B). Although ZFX ChIP-seq does not allow precise positioning of the bound ZFX, it appears that the summit of the ZFX peak is located in the NDR downstream from both the TSS and a bound RNA Polymerase II (RNAPII), just upstream of the first phased nucleosome (Fig. 6C,D). Indeed, >70% of ZFX peaks that have a summit near +240 bp of the TSS overlapped with NDRs called by NOMe-seq in MCF-7 and C4-2B cells (Supplemental Table S6). Although Figure 6D shows the pattern for all ZFX-bound promoters in MCF-7 cells, a similar pattern is also seen if the small subset of promoters bound by ZFX only in MCF-7 cells is analyzed (Supplemental Fig. S6).

## Discussion

We profiled ZFX binding sites genome-wide in kidney, colon, prostate, and breast cancer cell lines. Unlike many oncogenic TFs that bind to distal elements, ZFX binds to the majority of CpG island promoters that are active in cancer cells, and many genes with promoters bound by ZFX were down-regulated upon knockdown. Surprisingly, ZFX binds at +240 bp downstream from the TSS of ZFX-regulated promoters, in the open chromatin region between the TSS and the first downstream nucleosome. Genome-wide analyses of open chromatin and DNA methylation demonstrate that promoters bound by ZFX have a more accessible region, with lower levels of DNA methylation near the TSS and more highly phased nucleosomes downstream from the TSS, compared to promoters that are active but not bound by ZFX. Taken together, these findings support the hypothesis that ZFX may act as a transcription activator and play an important role in maintaining a nucleosome-free promoter region and/or in positioning nucleosomes at many CpG island promoters in the human genome.

In accordance with findings from previous studies (Fang et al. 2012, 2014a; Jiang et al. 2012a; Yang et al. 2014, 2015), we found that the top categories of genes affected by ZFX knockdown are related to the cell cycle, to the DREAM complex (which contains E2F family members), and/or to genes regulated by E2F family members (Supplemental Fig. S7). For example, cell division cycle 27 homolog (*CDC27*), a component of the anaphase promoting complex/cyclosome that ubiquitinates Cyclin B (Lee and Langhans 2012), and MutL homolog 3 (*MLH3*), which is implicated in maintaining DNA replication and mismatch repair (Lipkin et al. 2000), both have a ZFX binding site downstream from the TSS, and their expression levels are decreased upon ZFX knockdown in both MCF-7 and C4-2B. Thus, our results support the previous studies that ZFX expression is linked to cell proliferation. We also mapped ZFX binding sites in human normal prostate epithelial cells (PrEC) (Supplemental Fig. S8; Supplemental Table S3G). Although the ZFX binding pattern in normal prostate cells is very similar to the ZFX binding pattern in the prostate cancer cell line, the ChIP-seq peaks in PrEC were considerably smaller, suggesting that high ZFX expression in cancer cells may result in stronger binding and higher expression of genes involved in cell proliferation.

### What distinguishes a “functional” bound ZFX from a “nonfunctional” bound ZFX?

Although ZFX binds to approximately 8000–9000 promoters in a given cell type, siRNA-mediated knockdown of ZFX resulted in altered activity of only a subset of these promoters. Although the ZFX motif is enriched symmetrically ±240 bp from the TSS, our results suggest that ZFX acts as a transcriptional activator only when bound at +240 bp. However, not all promoters with a ZFX bound at +240 bp responded in the knockdown experiments. There are several possibilities that can explain why reduction of ZFX levels only affected a small percentage of promoters to which it is bound. First, it is possible that the incomplete knockdown of ZFX by siRNA treatment may have prevented the identification of all ZFX-regulated genes. In the future, knockout of ZFX by CRISPR/Cas9 could be performed to determine if a larger set of ZFX-regulated genes is identified upon complete removal of ZFX from the cell. It is also possible that cobinding of ZFX with other TFs is required for ZFX to regulate transcription. Finally, there is the possibility that other TFs are functionally redundant with ZFX. We tested the possibility that ZNF711, a TF that shares high homology and a similar DNA binding motif to ZFX, can substitute for ZFX. Indeed, ZNF711 binding sites are shared by ZFX binding sites, and we identified several hundred additional ZFX-bound target genes that are down-regulated upon knockdown of both ZFX and ZNF711; perhaps complete loss of both proteins is required to observe the full effect of ZFX on the transcriptome.

### How does ZFX regulate transcription of CpG island promoters from a downstream position?

There are two main types of transcriptional regulatory elements, promoters and enhancers. Unlike enhancers, which are located far from a TSS, are cell-type specific, and are closely linked to cellular identity (Rhie et al. 2014, 2016), promoter elements are crucial for basal transcription of genes. The majority of human promoters are classified as CpG island promoters; these promoters are generally active in most cell types (Deaton and Bird 2011). Interestingly, ZFX is bound to most of the active CpG island promoters in a given cell. Other TFs have been shown to preferentially bind to CpG island promoters (Rozenberg et al. 2008; Jaeger et al. 2010; Landolin et al. 2010; Blattler et al. 2013). However, these CpG island-binding TFs tend to bind upstream of the TSS (Cao et al. 2011), whereas ZFX binds 240 bp downstream from +1.

Comparison of the binding patterns of ZFX with RNAPII and H3K4me3 revealed that the bound ZFX is slightly downstream from the RNAPII signal and slightly upstream of the downstream peak of H3K4me3 signal (Fig. 6D). Although it is possible that ZFX regulates release of a paused RNAPII, factors implicated in this process are usually bound at +30 to +40 bp relative to the TSS (Krumm et al. 1995; Shao and Zeitlinger 2017). It is unlikely that ZFX is involved in splicing, since the binding site can be in the first exon or at various places within the first intron, depending on the size of the first exon. Moreover, RNAPII and H3K4me3 signals are more enriched at ZFX-bound promoters than at promoters not bound by ZFX (Supplemental Fig. S9); these findings are consistent with a role for ZFX in transcriptional (not post-transcriptional) regulation. ZFX does appear to be uniquely placed in relation to the phased nucleosomes located downstream from the TSS, and ZFX-bound promoters have a more open region near the start site than do promoters that are active but not bound by ZFX. Therefore, we postulate that perhaps ZFX is involved in creating a nucleosome-depleted region in CpG island promoters by recruiting the transcription preinitiation complex and/or in positioning the downstream nucleosomes (Fig. 6E).

In conclusion, we profiled ZFX binding sites genome-wide in kidney, colon, prostate, and breast cancer cells and found that ZFX may function as a transcriptional activator, regulating as many as 60% of active CpG island promoters. Because tumor cells require abnormally high levels of transcription for their inappropriate proliferation and survival, increased overall transcription mediated by ZFX may explain why this TF has been correlated with poor prognosis for a variety of human cancers (Jiang and Liu 2015; Li et al. 2015; Yang et al. 2015; Yan et al. 2016). Future studies will focus on testing the hypothesis that ZFX contributes to overall high levels of transcription via a role in maintaining the large NDR found at the ZFX-bound promoters. Our demonstration that ZNF711, a TF highly related to ZFX, has a similar binding pattern suggests that we may have identified a new class of regulatory TFs. Further characterization of these TFs and their role in gene regulation will provide important new insights into transcription, chromatin structure, and the regulation of the cancer transcriptome.

## Methods

### Cell culture

The human kidney HEK293T (ATCC# CRL-3216), colon HCT116 (ATCC# CCL-247), and breast cancer MCF-7 (ATCC# HTB-22) cells were obtained from ATCC (https://www.atcc.org/). The human prostate cancer C4-2B cells were obtained from ViroMed Laboratories. The human normal prostate epithelial cells (PrEC) were obtained from Lonza (Cat# CC-2555). The corresponding medium of each cell line (DMEM for HEK293T, McCoy's 5A for HCT116, RPMI 1640 for C4-2B, DMEM for MCF-7) was supplemented 10% fetal bovine serum (Gibco by Thermo Fisher Scientific) and 1% penicillin and streptomycin at 37°C with 5% CO_2_. PrEC cells were grown with PrEGM Bullet Kit (Prostate Epithelial Cell Growth Medium with supplements), which were obtained from Lonza (Cat# CC-3166). All cell stocks except primary cells (PrEC) were authenticated at the USC Norris Cancer Center cell culture facility by comparison to the ATCC and/or published genomic criteria for that specific cell line; all cells are documented to be free of mycoplasma. Preauthentication was performed at Lonza for PrEC, and the first passage from the cultured cells was used for the ChIP assay.

### ChIP-seq

ZFX ChIP assays were performed in HEK293T, HCT116, C4-2B, MCF-7, and PrEC cells using a ZFX antibody (Cat# L28B6 Lot# 1, Cell Signaling Technology) according to ENCODE standards (Blattler et al. 2014). The ZFX antibody was validated using siRNAs, followed by Western blots to demonstrate loss of the detected protein band (Supplemental Fig. S1). ZNF711 ChIP-seq experiments in MCF-7 cells were performed using antibodies from two different rabbits that were generated against ZNF711 amino acids 1–358; these antibodies have been previously used in ChIP-seq and were provided by Dr. Kristian Helin (Kleine-Kohlbrecher et al. 2010). H3K4me3 and RNAPII ChIP-seq experiments in C4-2B cells were performed using antibodies from Cell Signaling Technology (Cat# 9751S) for H3K4me3 and BioLegend (Cat# 664906) for RNAPII. Each ZFX/ZNF711 ChIP-seq experiment in cancer cells was performed using two biological replicates, and ChIP-seq libraries were sequenced on an Illumina HiSeq. All ChIP-seq data were mapped to hg19 and peaks were called using MACS2 (Zhang et al. 2008) with the IDR tool (https://github.com/nboley/idr) after preprocessing data with the ENCODE3 ChIP-seq pipeline (https://www.encodeproject.org/chip-seq/). ZFX and ZNF711 binding sites are listed in Supplemental Tables S3 and S5. A detailed description of ChIP-seq analyses can be found in Supplemental Methods.

### Motif analyses

To discover de novo motifs enriched in the ChIP-seq peaks, we collected sequences of 20-bp windows of the ZFX peak summits and used MEME version 4.10.1 (Bailey and Elkan 1994) with a minimum motif width of 6 and a maximum motif width of 12, scanning both strands of the DNA sequences. The discovered motifs were very similar to the known motifs for ZNF711 and ZFX; AGGCCTAG motif found from HOMER (http://homer.ucsd.edu/homer/) (Heinz et al. 2010) was originally identified from ZNF711 ChIP-seq in SH-SY5Y (Kleine-Kohlbrecher et al. 2010) and ZFX ChIP-seq in mouse embryonic stem cells (Chen et al. 2008). Therefore, we used known motifs to scan ZFX and ZNF711 binding sites in four cell types using findMotifsGenome.pl script from HOMER to identify the enriched motifs and calculate the percentage of regions with the motifs (Fig. 2A). The motifs reported in Figure 2A are the enriched motifs (FDR <0.05) found in >50% of ZFX peaks (sequences of 20-bp windows of the ZFX peak summits) in each cell type. To further examine motif distribution in promoters, we compared the ZFX motif (AGGCCTAG), 10 randomly scrambled motifs having the same nucleotide composition as the ZFX motif, and the ETS motif (Supplemental Fig. S2).

### siRNA knockdown, RT-qPCR, and RNA-seq

For transient knockdown, cells were transfected in triplicate with 100 nM of siRNA oligonucleotides targeting human ZFX (Cat# L006572000005), ZNF711 (Cat# L008444020005), or control oligonucleotides (Cat# D0018101005) using SMART pool DharmaFECT transfection reagent 3 (Cat# T200301) for C4-2B and reagent 1 (Cat# T200101) for MCF-7 (Dharmacon). Cells were incubated for 24 h and transfected again with the same concentration of siRNAs, and the incubation was continued for an additional 24 h. RNA was extracted using TRIzol reagent (Cat# 15596-018, Thermo Fisher Scientific) following the manufacturer-suggested protocol. cDNA was synthesized using the SuperScript VILO cDNA Synthesis Kit (Cat# 11754-050, Life technologies). RNA-seq libraries were made using KAPA Stranded mRNA-Seq Kit with KAPA mRNA Capture Beads (Cat# KK8421, Kapa Biosystems) and sequenced on an Illumina HiSeq. To remove batch effects, matched controls and knockdown samples were prepared and sequenced at the same time. Differentially expressed genes were selected using the Gene Specific Algorithm from Partek Flow software with the upper quartile normalization method (Partek Inc.). An FDR cutoff of 0.05 was used to select statistically significantly differently expressed genes. Differentially expressed genes with absolute fold change >1.5 are listed in Supplemental Table S4.

### NOMe-seq

The NOMe-seq method is a combination of the genome-wide identification of open chromatin regions plus whole-genome bisulfite sequencing (to identify methylated DNA). The first step of the method is based on the treatment of chromatin with the M.CviPI methyltransferase. This enzyme methylates Cs in the context of GpC dinucleotides. GpC^m^ does not occur in the human genome (the vast majority of DNA methylation in the human genome is at CpG dinucleotides, not GpC dinucleotides) and therefore there is no endogenous background of GpC^m^. The enzyme can only methylate GpC dinucleotides that are accessible in the context of chromatin, i.e., not protected by nucleosomes or other proteins that are tightly bound to the chromatin. The second step of the method involves bisulfite treatment of the M.CviPI-methylated chromatin, which converts unmethylated Cs to Ts. This allows the distinction of GpC from GpC^m^ and CpG from C^m^pG. Using this method, NDRs are defined as regions having increased GpC^m^ methylation over background (i.e., they are in open regions and thus were methylated by the M.CviPI enzyme) that are at least 140 bp in length. Because the bisulfite treatment also allows the distinction of CpG from C^m^pG, the endogenous methylation status of the genome can also be obtained in the same sequencing reaction. It is important to note that in contrast to the induced GpC^m,^ which represents nucleosome-free, open chromatin that is available for TF binding, the endogenous C^m^pG represents nucleosome-bound chromatin that is not available for TF binding. Open chromatin is expected to have high levels of GpC^m^ but low levels of C^m^pG; thus, each of the two separate methylation analyses serve as independent (but opposite) measures that should provide matching chromatin designations (open versus closed). C4-2B NOMe-seq data were generated as previously described (Rhie et al. 2018) and sequenced using an Illumina HiSeq 2000 PE 100 bp to produce FASTQ files. FASTQ files of MCF-7 NOMe-seq data were obtained from GSE57498 (Taberlay et al. 2014). Each FASTQ file was aligned to a bisulfite-converted genome (hg19) using BSMAP (Xi and Li 2009) and processed as previously described (Rhie et al. 2018). To identify the methylation status of CpG sites (in all HCG trinucleotides) and GpC sites (in all GCH trinucleotides) from the BAM file, the Bis-SNP (Liu et al. 2012) program was used and the Bis-tools was used to visualize DNA methylation and accessibility signals (https://github.com/dnaase/Bis-tools) (Lay et al. 2015). For identification of NDRs (Supplemental Table S6), the findNDRs function in the aaRon R package was used (https://github.com/astatham/aaRon).

### Gene Set Enrichment Analysis and Gene Ontology analysis

Differentially expressed genes upon ZFX knockdown are selected using FDR cutoff 0.05 and fold change cutoff 1.5 in C4-2B cells (Supplemental Table S4), and genes bound by ZFX were selected for Gene Set Enrichment Analysis (GSEA) and Gene Ontology (GO) analysis. The differentially expressed genes were used to identify enriched gene sets using the GSEA tool (Subramanian et al. 2005). Hypergeometric test was used to calculate *P*-value, and false discovery rate (*Q*-value) <0.05 was used to select significantly enriched gene sets. The same differentially expressed genes were analyzed for enrichment in particular GO categories using the TopGO program (https://bioconductor.org/packages/release/bioc/html/topGO.html). A Fisher's exact test was performed, and an adjusted *P*-value cutoff 0.05 was used to select statistically significantly enriched GO categories (Supplemental Figs. S7, S8E).

## Data access

All ChIP-seq, RNA-seq, and NOMe-seq data generated in this study have been submitted to the NCBI Gene Expression Omnibus (GEO; http://www.ncbi.nlm.nih.gov/geo/) under accession number GSE102616. Access to other publicly available data sets from GEO or ENCODE (The ENCODE Project Consortium 2012; Sloan et al. 2016) used in this study is detailed in Supplemental Table S1.

## Supplementary Material

Supplemental Material
