# Supplementary material for: ZFX acts as a transcriptional activator in multiple types of human tumors by binding downstream from transcription start sites at the majority of CpG island promoters
Source: Genome Res. 2018 Mar;28(3):310–20. doi: 10.1101/gr.228809.117 (PMC5848610; doi:10.1101/gr.228809.117)
Supplement: Supplemental Material [file supp_28_3_310__index.html]

ZFX acts as a transcriptional activator in multiple types of human tumors by binding downstream from transcription start sites at the majority of CpG island promoters — Supplemental Material 

# ZFX acts as a transcriptional activator in multiple types of human tumors by binding downstream from transcription start sites at the majority of CpG island promoters

## Supplemental Material

- Supplemental\_Fig\_S1.pdf
- Supplemental\_Fig\_S2.pdf
- Supplemental\_Fig\_S3.pdf
- Supplemental\_Fig\_S4.pdf
- Supplemental\_Fig\_S5.pdf
- Supplemental\_Fig\_S6.pdf
- Supplemental\_Fig\_S7.pdf
- Supplemental\_Fig\_S8.pdf
- Supplemental\_Fig\_S9.pdf
- Supplemental\_Methods.pdf
- Supplemental\_Table\_S1.xlsx
- Supplemental\_Table\_S2.xlsx
- Supplemental\_Table\_S3.xlsx
- Supplemental\_Table\_S4.xlsx
- Supplemental\_Table\_S5.xlsx
- Supplemental\_Table\_S6.xlsx
