# Supplementary material for: ZFX acts as a transcriptional activator in multiple types of human tumors by binding downstream from transcription start sites at the majority of CpG island promoters
Source: Genome Res. 2018 Mar;28(3):310–20. doi: 10.1101/gr.228809.117 (PMC5848610; doi:10.1101/gr.228809.117)
Supplement: Supplemental Material [file supp_gr.228809.117_Supplemental_Fig_S1.pdf]

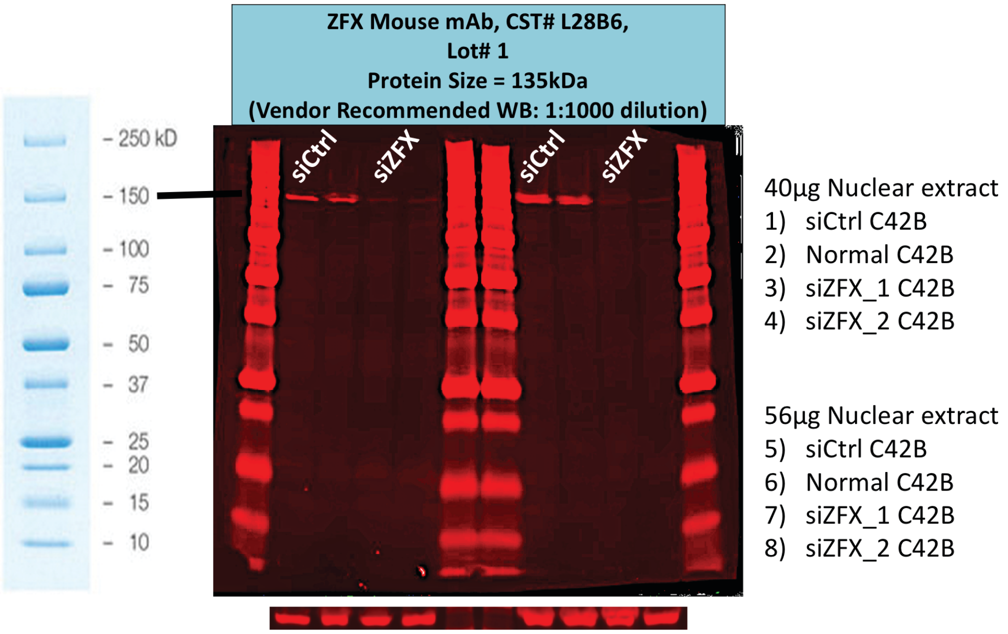

**Nucleoporin p62, BD Transduction Laboratories cat# 610497, Lot# 77287, Protein Size = 62kDa**	**WB**
**mouse monoclonal IgG, 2.5ug/uL**	**2.5ug/10000uL**

Supplemental Figure S1. ZFX antibody validation. Duplicate ZFX siRNA treatments of C42B cells were performed; 40ug of nuclear extract was analyzed on the left side of the gel and 56ug of nuclear extract was analyzed on the right side of the gel. A significantly reduced signal in the siZFX lanes can be seen using both amounts of nuclear extract. The Nucleoporin p62 antibody was used as a loading control; the p62 western blot image is also shown at the bottom.
