# Supplementary material for: ZFX acts as a transcriptional activator in multiple types of human tumors by binding downstream from transcription start sites at the majority of CpG island promoters
Source: Genome Res. 2018 Mar;28(3):310–20. doi: 10.1101/gr.228809.117 (PMC5848610; doi:10.1101/gr.228809.117)
Supplement: Supplemental Material [file supp_gr.228809.117_Supplemental_Fig_S2.pdf]

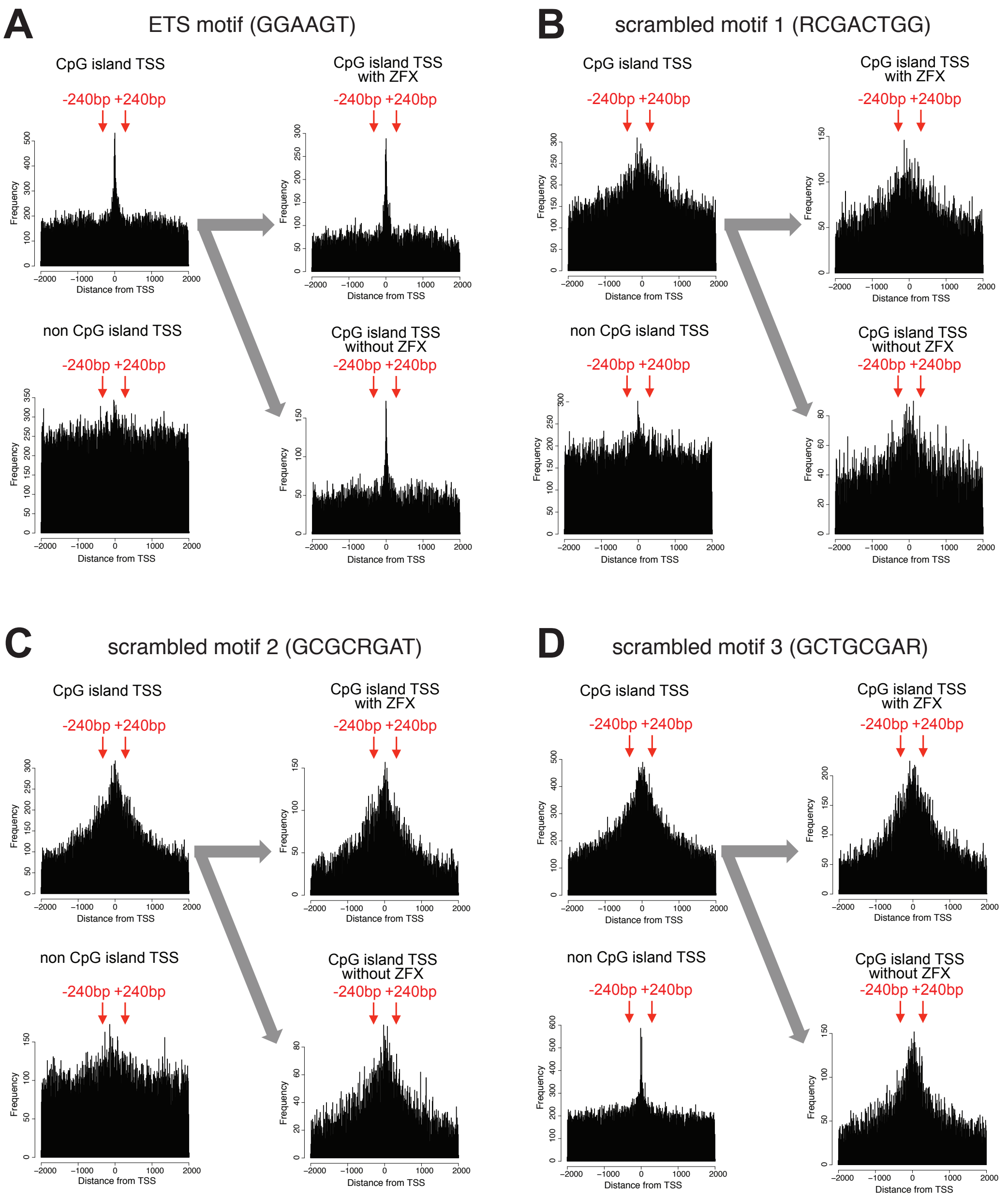

Supplemental Figure S2. Scrambled ZFX motifs are not enriched at ±240bp of the TSS. Shown is the frequency at which the ETS motif GGAAGT, as an example of a known motif for a TF other than ZFX that binds to promoter regions (A), scrambled ZFX motif 1 (B), scrambled ZFX motif 2 (C), or scrambled ZFX motif 3 (D) are located ±2kb from the TSS of CpG island promoters, of non-CpG island promoters, of CpG island promoters bound by ZFX, and of CpG island promoters not bound by ZFX.
