# Supplementary material for: ZFX acts as a transcriptional activator in multiple types of human tumors by binding downstream from transcription start sites at the majority of CpG island promoters
Source: Genome Res. 2018 Mar;28(3):310–20. doi: 10.1101/gr.228809.117 (PMC5848610; doi:10.1101/gr.228809.117)
Supplement: Supplemental Material [file supp_gr.228809.117_Supplemental_Fig_S3.pdf]

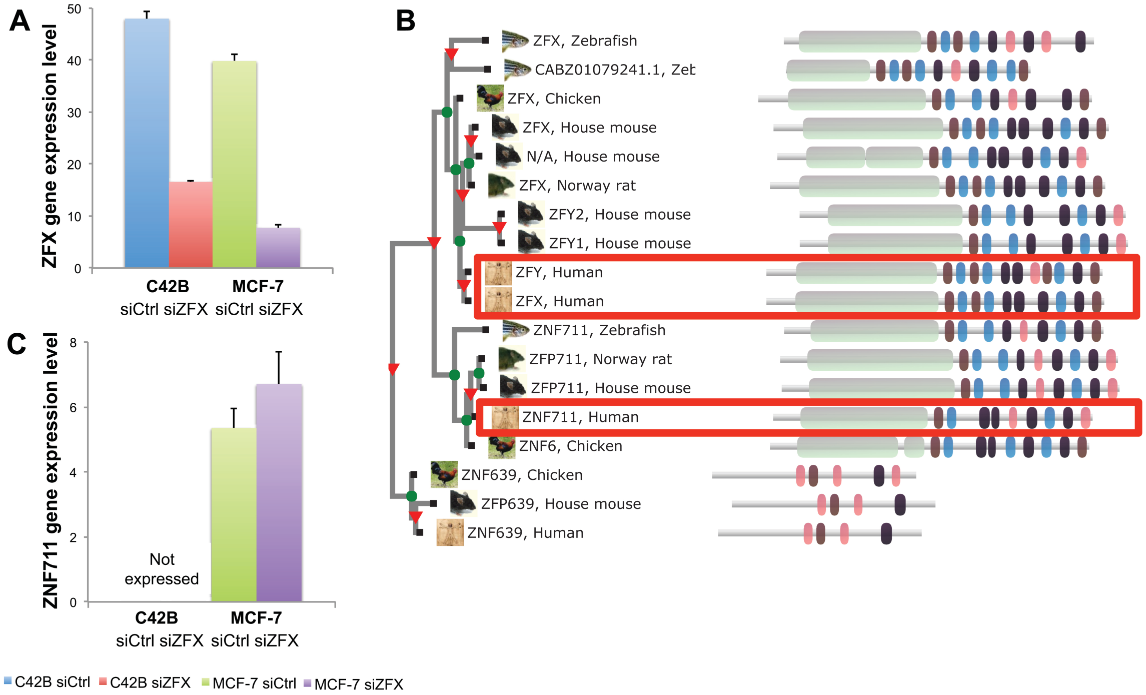

Supplemental Figure S3. ZFX and ZNF711 may have redundant functions. (A) Shown are expression levels of ZFX in C42B and MCF-7 upon ZFX knockdown. (B) Treefam gene tree analysis for the C2H2 ZNF family branch that includes ZFX. (C) Shown are expression levels of ZNF711 in C42B and MCF-7 cells upon ZFX knockdown.
