# Supplementary material for: ZFX acts as a transcriptional activator in multiple types of human tumors by binding downstream from transcription start sites at the majority of CpG island promoters
Source: Genome Res. 2018 Mar;28(3):310–20. doi: 10.1101/gr.228809.117 (PMC5848610; doi:10.1101/gr.228809.117)
Supplement: Supplemental Material [file supp_gr.228809.117_Supplemental_Fig_S4.pdf]

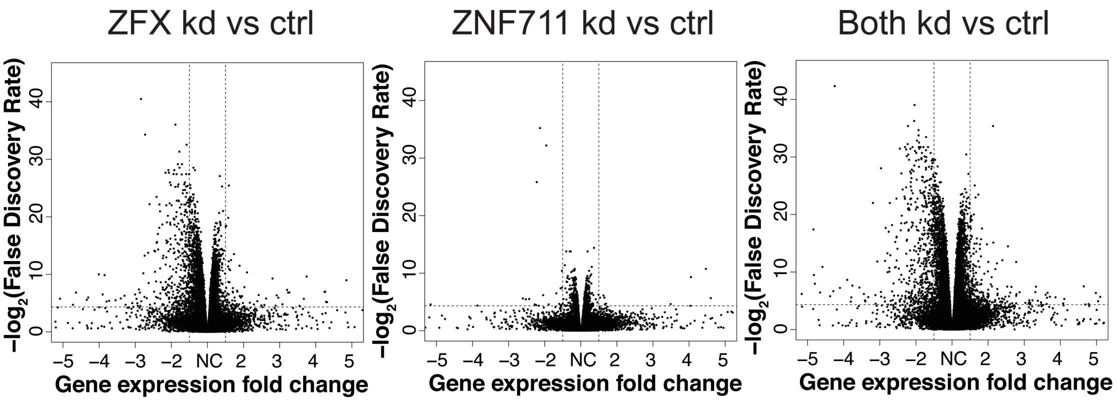

Supplemental Figure S4. Knockdown of ZFX and ZNF711 in HEK293T cells. Shown are volcano plots demonstrating differential gene expression after knockdown of ZFX, ZNF711, or both TFs in HEK293T cells (RNA-seq data are courtesy of W. Zhu and P.J. Farnham).
