# Supplementary material for: ZFX acts as a transcriptional activator in multiple types of human tumors by binding downstream from transcription start sites at the majority of CpG island promoters
Source: Genome Res. 2018 Mar;28(3):310–20. doi: 10.1101/gr.228809.117 (PMC5848610; doi:10.1101/gr.228809.117)
Supplement: Supplemental Material [file supp_gr.228809.117_Supplemental_Fig_S5.pdf]

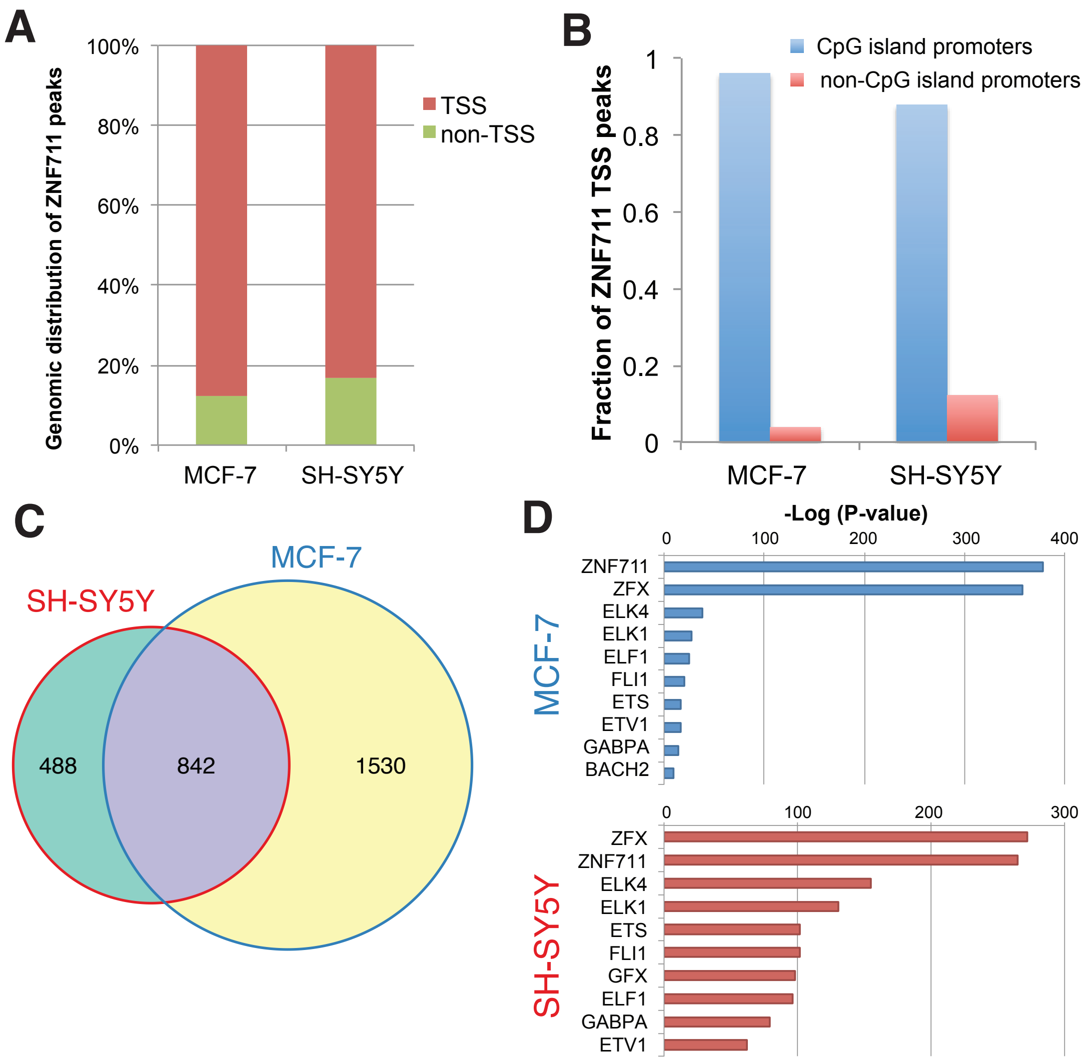

Supplemental Figure S5. Further characterization of ZNF711 binding sites using ChIP-seq datasets in MCF-7 and human neuroblastoma SH-SY5Y generated with the same ZNF711 antibody. (A) Shown is a genomic distribution of ZNF711 binding sites (TSS vs non-TSS). (B) The fraction of ZNF711 binding sites located in CpG island promoters vs non-CpG island promoters is shown for MCF-7 and SH-SY5Y. (C) Shown is a comparison of ZNF711 binding sites in promoters between MCF-7 and SH-SY5Y. (D) Motifs, which are most enriched (top10) in ZNF711 binding sites in MCF-7 (top) and SH-SY5Y (bottom), respectively, are listed with -Log(P-value) of each motif, which is calculated using findMotifsGenome.pl script from HOMER.
