# Supplementary material for: ZFX acts as a transcriptional activator in multiple types of human tumors by binding downstream from transcription start sites at the majority of CpG island promoters
Source: Genome Res. 2018 Mar;28(3):310–20. doi: 10.1101/gr.228809.117 (PMC5848610; doi:10.1101/gr.228809.117)
Supplement: Supplemental Material [file supp_gr.228809.117_Supplemental_Fig_S6.pdf]

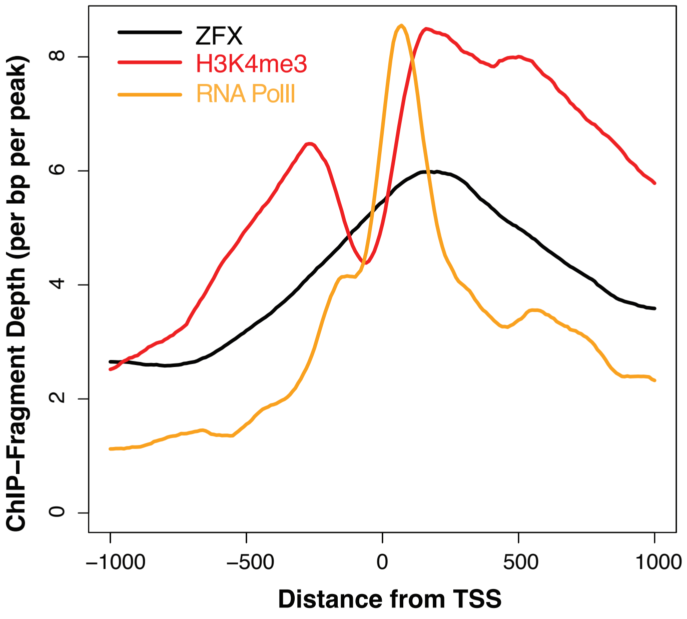

Supplemental Figure S6. MCF-7 cell type-specific ZFX binding sites are also located at downstream of TSS. Shown are the average ZFX (black), H3K4me3 (red), and RNA Polymerase II (orange) ChIP-seq signals ±1kb from the TSS of MCF-7 specific genes bound by ZFX (381 TSS sites which are bound by ZFX only in MCF-7).
