# Supplementary material for: ZFX acts as a transcriptional activator in multiple types of human tumors by binding downstream from transcription start sites at the majority of CpG island promoters
Source: Genome Res. 2018 Mar;28(3):310–20. doi: 10.1101/gr.228809.117 (PMC5848610; doi:10.1101/gr.228809.117)
Supplement: Supplemental Material [file supp_gr.228809.117_Supplemental_Fig_S7.pdf]

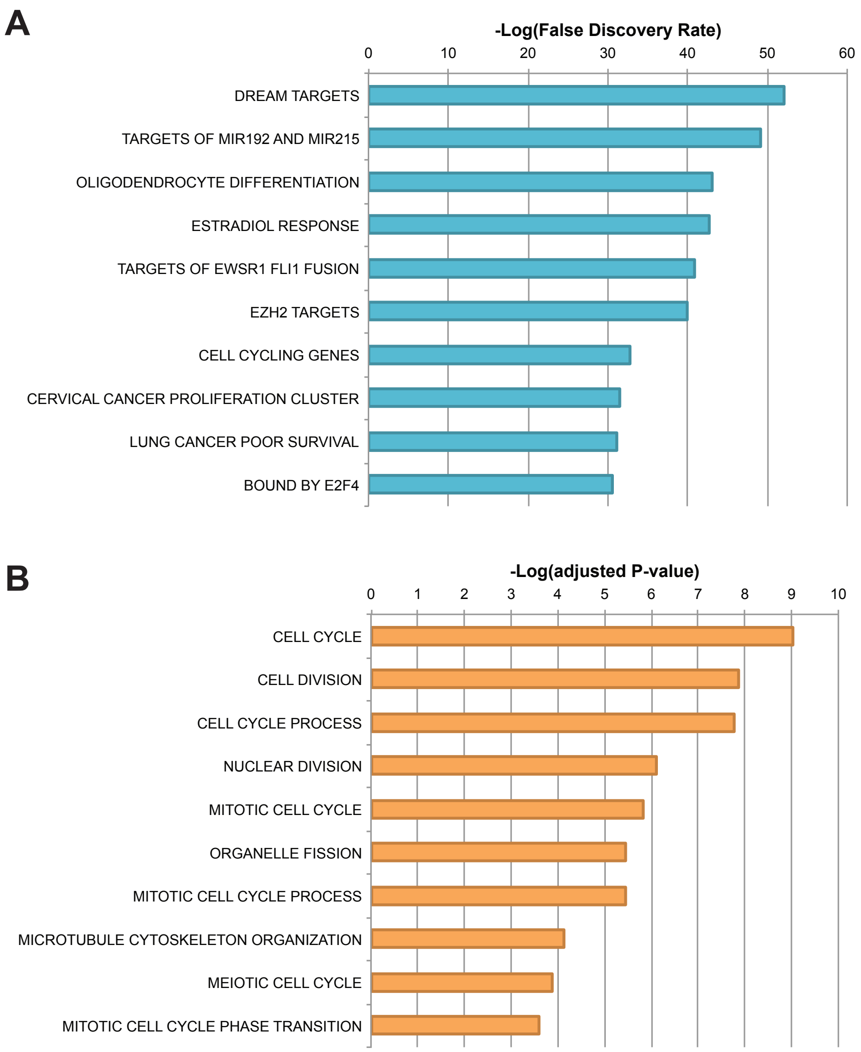

Supplemental Figure S7. Gene Set Enrichment Analysis (GSEA) and Gene Ontology (GO) analysis of ZFX-regulated genes. Shown are the top 10 gene sets/categories of ZFX-regulated genes from GSEA (A) and GO (B) analysis. The set of ZFX-regulated genes used were differentially expressed genes in siZFX- vs. siCtrl-treated C42B cells with promoters bound by ZFX (Supplemental Table S4).
