# Supplementary material for: ZFX acts as a transcriptional activator in multiple types of human tumors by binding downstream from transcription start sites at the majority of CpG island promoters
Source: Genome Res. 2018 Mar;28(3):310–20. doi: 10.1101/gr.228809.117 (PMC5848610; doi:10.1101/gr.228809.117)
Supplement: Supplemental Material [file supp_gr.228809.117_Supplemental_Fig_S8.pdf]

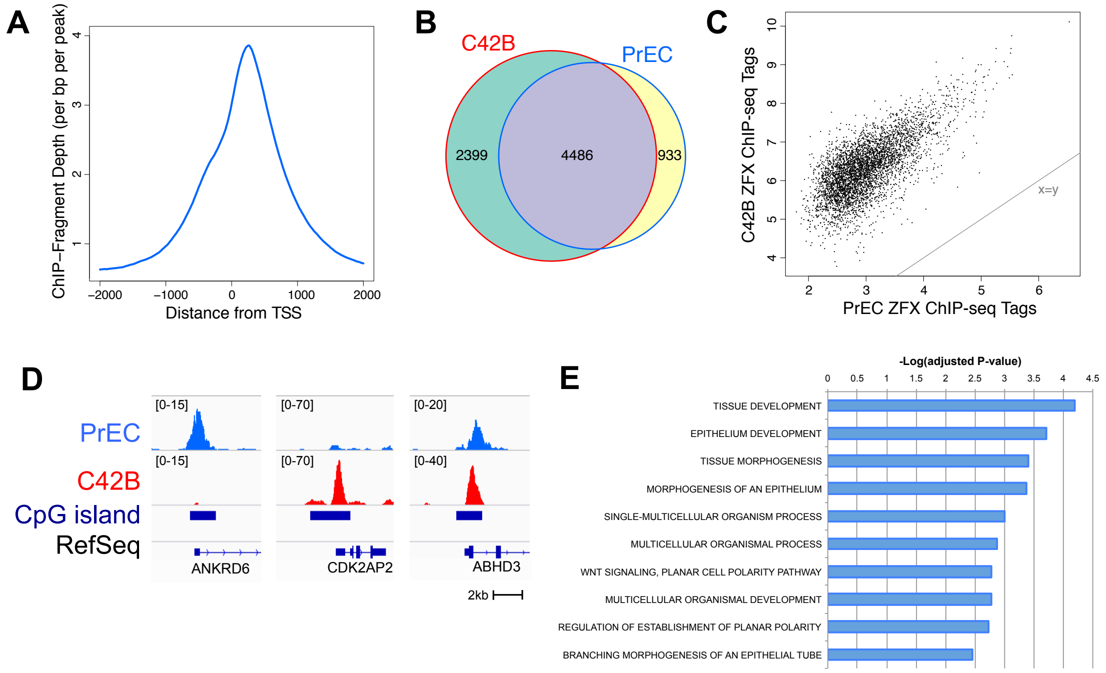

Supplemental Figure S8. ZFX binding sites in normal prostate epithelial cells (PrEC). (A) Shown is the average ZFX ChIP-seq signal ±2kb from the TSS of promoters bound by ZFX in PrEC. (B) Shown is a comparison of C42B and PrEC ZFX binding sites in promoter regions. (C) Shown is a scatter plot of the normalized PrEC ZFX vs. C42B ZFX ChIP-seq tags for the shared ZFX binding sites found in promoters. A gray line indicates x=y, a diagonal line. (D) Shown are examples of PrEC-specific (left), C42B-specific (center), and common ZFX binding sites located at CpG island promoters. (E) Shown are the top 10 categories that are enriched from GO (Gene Ontology) analysis of genes with PrEC-specific ZFX binding at their promoters.
