# Supplementary material for: ZFX acts as a transcriptional activator in multiple types of human tumors by binding downstream from transcription start sites at the majority of CpG island promoters
Source: Genome Res. 2018 Mar;28(3):310–20. doi: 10.1101/gr.228809.117 (PMC5848610; doi:10.1101/gr.228809.117)
Supplement: Supplemental Material [file supp_gr.228809.117_Supplemental_Fig_S9.pdf]

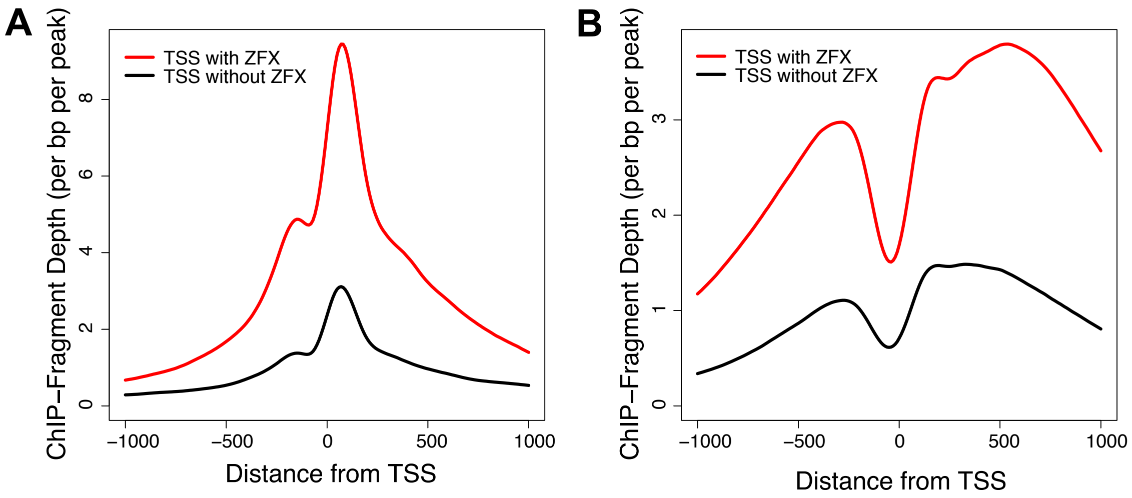

Supplemental Figure S9. RNAPII and H3K4me3 signals are more enriched at promoters bound by ZFX. Shown are the average RNAPII (A) and H3K4me3 (B) normalized ChIP-seq signals ±2kb from the TSS of promoters bound by ZFX (red) and promoters not bound by ZFX (black) in MCF-7 cells.
