# Supplementary material for: ZFX acts as a transcriptional activator in multiple types of human tumors by binding downstream from transcription start sites at the majority of CpG island promoters
Source: Genome Res. 2018 Mar;28(3):310–20. doi: 10.1101/gr.228809.117 (PMC5848610; doi:10.1101/gr.228809.117)
Supplement: Supplemental Material [file supp_gr.228809.117_Supplemental_Methods.pdf]

## Supplemental Methods

**Genomic distribution of ChIP-seq peaks.** To determine the genomic distribution of ZFX binding sites, the set of all TSS for known genes was obtained from Gencode (version 19, http://www.gencodegenes.org/releases/19.html) and promoters were defined as ±2kb windows from each TSS. CpG island locations were obtained using the UCSC Genome browser table tool (http://genome.ucsc.edu/). ChIP-seq datasets for H3K4me3, H3K27Ac, CTCF, and RNA Polymerase II were either generated in-house or obtained from ENCODE and GEO; see Supplemental Table S1. Bedtools (https://github.com/arq5x/bedtools2) were used to intersect genomic regions and the Vennerable R package was used to generate weighted Venn diagrams; ChowRuskey type Venn diagrams were used for comparison of more than three datasets (http://download.r-forge.r-project.org/src/contrib/Vennerable_3.0.tar.gz).

**Comparison of ZFX ChIP-seq peaks.** Using the DiffBind R package (Ross-Innes et al. 2012), ZFX ChIP-seq signals were normalized across replicates and cell types (setting minOverlaps to 2 in the dba.count function) at ZFX binding sites from 4 cell types. The correlation coefficient was calculated using the dba.plotHeatmap function.

**Comparison of ZNF711 ChIP-seq peaks.** ZNF711 ChIP-seq data in SH-SY5Y from GSM518560 was processed by Cistrome DB pipeline (http://cistrome.org/) and peaks were called using hg38. UCSC tool, liftOver was used to convert these peaks to re-align in hg19 for further analysis with ZNF711 ChIP-seq data in MCF-7 (hg19). ZNF711 ChIP-seq peaks in MCF-7 and SH-SY5Y were compared using TSS and CpG island annotation, which are described above.

**Average plots, Heatmaps, and scatter plots for ChIP-seq data.** ZFX, ZNF711, H3K4me3, and RNAPII ChIP-seq tags were normalized using the MakeTagDirectory script from HOMER

(http://homer.ucsd.edu/homer/) (Heinz et al. 2010). Normalized ChIP-seq tags at peaks, motifs, and promoters were used to make average plots, heatmaps, and scatter plots using the annotatePeaks.pl script from HOMER. The heatmap for Fig. 2I was generated by clustering normalized ChIP-seq tags and visualized using fastcluster and heatmap.2 R packages.

**Visualization of ChIP-seq and NOMe-seq data.** To visualize ChIP-seq and NOMe-seq data and generate the genome browser screenshots, the Integrative Genomics Viewer (IGV) (Thorvaldsdottir et al. 2013) was used.

**Western blots.** Nuclear extracts were prepared from HEK293T, HCT116, C42B and MCF-7 cells using Cell lysis buffer (5mM PIPES pH 8.0, 85mM KCl, Igepal 10µL/mL) and Nuclei lysis buffer (50mM Tris-Cl pH 8.1, 10mM EDTA, 1% SDS supplemented with protease inhibitor (Sigma-Aldrich, St. Louis, MO). Equal amounts of protein from whole cell extracts were separated by running on SDS-PAGE gels (Cat # 456-9025, Bio-Rad, Hercules, CA), and transferred to a nitrocellulose sheet (GE Healthcare, Buckinghamshire, UK). Membranes were preblocked with 1x TBST (1xTBS and 0.1% Tween-20) and 5% milk and incubated overnight with a diluted anti-ZFX (Cat # L28B6 Lot # 1, Cell Signaling Technology, MA, USA). On the next day, the membranes were washed and incubated with a corresponding secondary antibody, anti-mouse IgG, HRP-conjugated antibody (sc-2031) (Santa Cruz, Dallas, Texas). The membranes were scanned to detect bands for ZFX using the SuperSignal West Pico Chemiluminescent Substrate (Pierce, Rockford, IL, USA) and a ChemiDoc XRS + Imaging System with Image Lab (Bio-Rad, Hercules, CA). These membranes were also incubated with the nucleoporin control antibody (Cat # 610497, Lot # 77287, BD Transduction Laboratories, San Jose, CA, USA) for 1 hour at room temperature followed by the same secondary antibody incubation process, and scanned to detect bands for the control antibody; this served as a loading control (Supplemental Fig. S1).

## REFERENCES


Heinz S, Benner C, Spann N, Bertolino E, Lin YC, Laslo P, Cheng JX, Murre C, Singh H, Glass CK. 2010. Simple combinations of lineage-determining transcription factors prime cis-regulatory elements required for macrophage and B cell identities. *Molecular cell* **38**: 576-589.

Ross-Innes CS, Stark R, Teschendorff AE, Holmes KA, Ali HR, Dunning MJ, Brown GD, Gojis O, Ellis IO, Green AR et al. 2012. Differential oestrogen receptor binding is associated with clinical outcome in breast cancer. *Nature* **481**: 389-393.

Thorvaldsdottir H, Robinson JT, Mesirov JP. 2013. Integrative Genomics Viewer (IGV): high-performance genomics data visualization and exploration. *Brief Bioinform* **14**: 178-192.
